# Aripiprazole-Induced Oculogyric Crisis: A Pediatric Case Series and A Brief Narrative Review

**DOI:** 10.3390/children9010022

**Published:** 2021-12-29

**Authors:** Pia Bernardo, Alfonso Rubino, Claudia Santoro, Carmela Bravaccio, Marco Pozzi, Simone Pisano

**Affiliations:** 1 Department of Neurosciences, Pediatric Psychiatry and Neurology, Santobono-Pausilipon Children’s Hospital, 80120 Naples, Italy; pia.bernardo84@gmail.com; 2 Department of Neurosciences, Pediatric Neurology, Santobono-Pausilipon Children’s Hospital, 80129 Naples, Italy; rubinoalfonso80@gmail.com; 3 Neurofibromatosis Referral Center, Department of Woman, Child, General and Specialized Surgery, Università degli Studi della Campania “Luigi Vanvitelli”, 80138 Naples, Italy; dr.claudiasantoro@gmail.com; 4 Clinic of Child and Adolescent Neuropsychiatry, Department of Mental and Physical Health, and Preventive Medicine, Università degli Studi della Campania “Luigi Vanvitelli”, 80138 Naples, Italy; 5 Department of Translational Medical Sciences, Federico II University, 80138 Naples, Italy; carmela.bravaccio@unina.it; 6 Scientific Institute IRCCS Eugenio Medea, 23842 Bosisio Parini, Italy; marco.pozzi@lanostrafamiglia.it

**Keywords:** aripiprazole, extrapyramidal effects, pediatric, oculogyric crisis

## Abstract

Oculogyric crisis (OGC) represent an unusual type of dystonic movement disorder, usually reported as an adverse event of antipsychotic drugs, with acute or tardive onset, likely due to a functional disruption of dopaminergic neurotransmission. It is seldom reported in children with aripiprazole, an atypical antipsychotic commonly used in youths. In this manuscript, we report on a case series of three pediatric patients and provide a brief narrative review of the literature, in order to increase the awareness of clinicians and to foster future research in this area.

## 1. Introduction

Aripiprazole is an atypical antipsychotic often used in youths with tic disorders, psychosis spectrum disorder, bipolar disorder and irritability in the context of autism spectrum disorder. Within the group of atypical antipsychotics, aripiprazole shows a relatively benign safety profile (e.g., lower metabolic impact, mild effect on cardiovascular parameters) [1], although the reported rate of extrapyramidal side effects is substantial [2,3]. Pediatric populations may be more susceptible to aripiprazole-induced extrapyramidal symptoms (EPS) than adults because of differences in terms of pharmacokinetics, pharmacodynamics, and dopaminergic receptor density, which is inversely proportional to age [2]. However, in both pediatric and adult populations, the incidence of EPS can vary substantially, often depending on differences in antipsychotic dose.

Oculogyric crisis (OGC) is a rare movement disorder, characterized by a sustained, conjugate, usually upwards deviation of the eyes, lasting minutes to hours [4,5]. Originally described in subjects with postencephalitic Parkinsonism, OGCs have been recognized in association with several conditions as hereditary and sporadic movement disorders or focal brain lesions [5]. However, in most cases, OGC is a drug-induced adverse event with acute or tardive, yet abrupt, onset [6], typically attributable to a functional impairment of dopaminergic neurotransmission [5]. Antipsychotic drugs belong to the class mostly involved in causing OGC. OGCs are often accompanied by increased blinking of the eyes, ocular pain, backward and lateral flexions of the neck, blepharospasm, wide open mouth and tongue protrusion [7]. Psychiatric symptoms, or their exacerbation, may also occur during episodes of OGC [8,9]. OGC is seldom reported in children during treatment with aripiprazole, although it is commonly used in youths. Herein, we report on three pediatric cases of OGC induced by aripiprazole and narratively review the existing literature (Table 1).

## 2. Case Presentations

### 2.1. Case 1

#### Patient Information

The first patient, a 13-year-old female, suffered from obsessive compulsive disorder (OCD) and anxiety disorder, with psychotic symptoms (persecutory ideas, visual hallucinations, marked social anxiety, states of psychomotor agitation). In her pharmacological anamnesis she had already taken several drugs, including lamotrigine and risperidone, which was discontinued for weight gain. Subsequently, she achieved good symptom control with valproic acid (VPA) 25 mg/kg/day, fluoxetine 20 mg/day and aripiprazole in association. Three months after an aripiprazole dose increase from 10 to 15 mg/day, she experienced a single episode of OGC, characterized by persistent upward deviation of the eyes, associated with pain and anxiety, profuse sweating, facial flushing, increase in obsessive thoughts and panic, lasting for 30–40 min approximately. A video-EEG and brain MRI were normal. After aripiprazole dose reduction to 10 mg/day, the patient presented no further episodes in the following 18 months, and her psychiatric symptoms were well controlled.

### 2.2. Case 2

#### Patient Information

The second patient, an 11-year-old male with ASD, treated with aripiprazole 10 mg/day, presented several episodes of OGC, characterized by gaze deviation up and right, and head deviation to the right. Episodes began one month after the introduction of aripiprazole, they lasted about 10 min each and had a frequency of two to three episodes per month, with spontaneous resolution. An EEG had been performed, showing no epileptiform abnormalities. Aripiprazole was reduced to 7.5 mg/day and the patient had no further episodes during the following 12 months.

### 2.3. Case 3

#### Patient Information

The third patient, a 14-year-old male, suffered from ASD and intellectual disability and began aripiprazole treatment for persistent and disabling irritability, emotional and behavioral dysregulation. He was taking VPA 20 mg/kg/day for a well-controlled focal occipital epilepsy. His EEG was stable, with epileptic abnormalities on the left occipital regions during sleep. The first OGC occurred around two weeks after increasing aripiprazole from 7 mg to the final titration step of 10 mg/day. The patient experienced upward deviation of the eyes, accompanied by eye pain, anxiety, headache and back head bending. An EEG and brain MRI were performed, showing no new abnormalities. Given a worsening in disruptive behaviors, he then gradually switched from aripiprazole to risperidone. After a few weeks he presented two more episodes of OGC. Trihexyphenidyl was added, and no episodes happened during six months of follow up.

### 2.4. Narrative Review

We searched non-systematically the English literature for “OGC and aripiprazole”, “OGC and antipsychotic”, “Ocular dystonia and aripiprazole” by using PubMed, Embase and Sciencegate. We identified ten articles describing eleven cases of OCG induced by aripiprazole (alone or in combination with other antipsychotics), summarized in Table 1. The subjects described were young adults (nine) or children (two). No specific psychiatric condition emerged as a determinant for the susceptibility to develop OGC. In addition, more than half of the cases showed other psychiatric symptoms or exacerbation of pre-existing symptoms in coincidence with episodes of OCG. In most subjects described, the OGCs had acute onset within 1 month of therapy initiation or up-titration. In one case, a subject with Down syndrome, OGC occurred 9 months after starting aripiprazole therapy [10]. Only in one case OCG occurred after more than 1 year of therapy. Furthermore, in this patient it was not sufficient to reduce the aripiprazole dosage, but discontinuation was necessary [11]. The OGCs were characterized by lifting or deviation of the eyes, lasting from 5 min to 2 h. Three of the subjects described also used other antipsychotics: olanzapine, risperidone, quetiapine [6,12,13]. In one pediatric case, aripiprazole was added to methylphenidate [14]. In two cases the onset of the OGC preceded the use of aripiprazole and occurred with olanzapine and paliperidone [13]. 

In all cases except one, the resolution of symptoms occurred after the discontinuation of aripiprazole and the adjunct of benzodiazepines or anticholinergics [15,16,17,18]. In one case, despite stopping all psychotropic medications for 3 months, recurrent episodes of OGC persisted for 7 months. The authors described this phenomenon as a long-lasting dystonia that included tardive and acute, persistent dystonia [13].

## 3. Discussion

We reported here three cases of OGC following treatment with aripiprazole in pediatric patients. We found a substantial phenomenological overlap with adult cases (>18 years), with the exception that, in our cases, OGCs happened without a previous exposure to another antipsychotic, thus excluding the possibility of a cumulative pharmacological effect [2,6,12,13]. Two of our patients were on combined treatment with valproic acid and fluoxetine, but they are uncommonly associated with OGCs.

All our patients presented with OGC from a few weeks to several months after the initiation of aripiprazole, often after dose increase. The OGCs reported had the typical characteristics, such as ocular pain and upward gaze deviation, in all patients. In one patient, OGCs were also accompanied with “stereotypical paroxysmal psychiatric symptoms” with increase of anxiety, profuse sweating, face flushing, obsessive thoughts; another patient reported headache. 

These clinical features underline how the differential diagnosis may be challenging, especially in the pediatric age group and in neurodevelopmental disorders, where a higher frequency of other movement disorders is observed, as compared to adults [2]. Indeed, ocular deviation movements must be evaluated critically during the diagnostic assessment, to rule out epileptic seizures, oculogyric tics as part of tic disorders, or clinical manifestations of neurometabolic disorders [5]. A thorough interview with caregivers, and, when possible, with the patient, prolonged observation possibly with video recording at home, and, when in doubt, a video EEG recording are useful tools to achieve a good diagnostic procedure. 

Finally, in our small case series, while in the first two cases (1 and 2) there was a complete remission of the OGCs following aripiprazole dose reduction, suggesting the clinical manifestation was a dose-dependent phenomenon [2,10], in the last patient (3) we observed persistence of the dystonic episodes despite the switch to another second-generation antipsychotic drug. The persistence of OGC after discontinuation or switch to another antipsychotic drug has been previously reported [6,13,14], emphasizing the role of individual susceptibility, for example the hypothesized dopaminergic receptor density, as a substrate for developing OGCs.

The underlying pathophysiology of OCG is not adequately characterized; dopamine dysregulation with an hypodopaminergic state is likely a requirement. Aripiprazole acts as a partial antagonist on the mesolimbic pathway stimulating presynaptic D2 receptors reducing dopamine release and as a partial agonist on D2 at the postsynaptic level, producing limited effects on dopamine in the nigrostriatal pathway [19]. It has been supposed that this peculiar dopaminergic profile could be associated with a lower rate of EPS. However, considering that aripiprazole lacks anticholinergic effect and that evidence support an intrinsic activity of the drug consistent with a functional D2 striatal blockage, the incidence of EPS during treatment with aripiprazole is not negligible [20]. Therefore, aripiprazole-related OGCs must be considered within the frame of dystonic reaction, requiring an acute change in dopamine regulation of a previously intact system with a lack of presynaptic dopaminergic degeneration [4].

The present report should raise awareness among clinicians for this relevant possible adverse event, that may happen also with the use of aripiprazole in pediatric patients, not only with typical or more antidopaminergic antipsychotics. We highlight the intrinsic difficulty in obtaining a detailed evaluation of OGCs in patients with neurodevelopmental disorders. Future research in the field should emphasize neurobiological dysfunctions as the basis of EPS/OGC in younger patients, also using neurophysiological and neuroimaging diagnostic tools. 

## Figures and Tables

**Table 1:** Review of cases of aripiprazole-related OGC reported in the literature.

Author	Sex/Age	Diagnosis	Clinical Features	Associated Movements/Psychiatric Symptoms	Dose	Duration of Treatment	Other Drugs	Management Strategy	Resolution (Y/N)
Canol T et al., 2020	F/14	Bipolar disorder	Description: crossing vertical deviation and continuous upward position of her eyes Duration: 2 h Frequency: once a week		15 mg	2 years	Quetiapine 150 mg	Discontinued	Y
Mercan Isik C et al., 2020	F/11	Attention deficit hyperactivity disorder	Description: upper visual fixation		2.5 mg	3rd day	Methylphenidate 36 mg/day	Biperiden 1 × 5 mg	Y
Suthar N et al., 2018	M/19	Obsessive compulsive disorder	Description: up-rolling of the eye balls and blurring of vision Duration: 5–10 min Frequency: 5–7 times in a day	tightening of the neck muscles with backward and right sided deviation of head along withpain/anxiety	10 mg	<1 months (5 days)	Fluoxetine 80 mg	Switch to promethazine	Y
Nebhinani N et al., 2017	F/22	Borderline personality disorder	Description: upward deviation and lateral fixation of eyes Duration: 15–20 min,		30 mg	<1 months (2 weeks)	No	Dose reduction to 20 mg, add on oftrihexyphenidyl	Y
Gardner DM et al., 2015	F/22	Schizoaffective disorder, bulimia and depression	Description: upward deviation of the eyes Duration: 20–60 min Frequency: 1–5 episodes/mo Recurrences: frequent 2 y, then infrequent 3 y		NA	NA (onset with other antipsychotics drugs)	Quetiapine and valproic acid	Add on of Niaspan^®^ formulation of niacin	Y
	F/16	Schizophrenia, intellectual disability, in DiGeorge Syndrome	Description: OGC Duration: 10 min Frequency: ~1/wk. Recurrences: 2–3 wk	discomfort and an inability to focus	10 mg	3 months	Risperidone 1 mg/die	Drug withdrawal	Y
Gupta et al., 2014	F/23	Obsessive compulsive disorder	Description: up-rolling of eyeballs Duration: 1–2 h Frequency: 7–8/times in a month	retrobulbar dull ache and stretching sensation.	30 mg	<1 months	Olanzapina 15 mg	Switch to escitalopram, propranolol, trihexyphenidyl	N
Rizzo et al., 2012	F/21	Tourette syndrome	Description: spasmodic upward deviations of the eyes Duration: 1–2 h Frequency of 3–4 times per week.	hyperextension of the cervical muscles/drowsy and markedly less active, but was able to follow commands	7.5 mg	<1 month (6 days)	Fluoxetine	Dose reduction to 2.5 mg	Y
Bhachech JT et al., 2012	F/28	Paranoid schizophrenia	Suddenly and multiple episodes of OGC		20 mg (70 kg)	<1 month (19 days)	Eszopiclone	Switch to promethazine	Y
Lim HK et al., 2008	M/23	Down syndrome, ID, schizophrenia paranoid type	Description: fixed upward gaze Duration: 1 h Frequency: 2–3 times per week	hyperextended neck of right cervical muscles, torticollis	10 mg/day	9 months	No	Dose reduction to 5 mg/day; add on of lorazepam 1 mg/day, procyclidine 5 mg/day	Y
Fountoulakis KN et al., 2006	M/18	Tourette syndrome	Acute OGC	facial muscle spasm and torticollis	10 mg (0.15 mg/kg)	<1 month (3 days)	No	Add on of biperiden	Y

## Data Availability

The data supporting the findings of this study are private due to patients’ privacy, but they are available from the corresponding author.
